# Hydrogen Utilization Potential in Subsurface Sediments

**DOI:** 10.3389/fmicb.2016.00008

**Published:** 2016-01-26

**Authors:** Rishi R. Adhikari, Clemens Glombitza, Julia C. Nickel, Chloe H. Anderson, Ann G. Dunlea, Arthur J. Spivack, Richard W. Murray, Steven D'Hondt, Jens Kallmeyer

**Affiliations:** ^1^MARUM - Center for Marine Environmental Sciences, University of BremenBremen, Germany; ^2^Geomicrobiology Group, Institute of Earth and Environmental Science, University of PotsdamPotsdam, Germany; ^3^Center for Geomicrobiology, Department of Bioscience, Aarhus UniversityAarhus C, Denmark; ^4^Helmholtz-Centre Potsdam - GFZ German Research Centre for GeosciencesPotsdam, Germany; ^5^Department of Earth and Environment, Boston UniversityBoston, MA, USA; ^6^Graduate School of Oceanography, University of Rhode IslandKingston, RI, USA

**Keywords:** hydrogenase, tritium assay, deep biosphere, microbial activity, Lake Van, Barents Sea, Equatorial Pacific, Gulf of Mexico

## Abstract

Subsurface microbial communities undertake many terminal electron-accepting processes, often simultaneously. Using a tritium-based assay, we measured the potential hydrogen oxidation catalyzed by hydrogenase enzymes in several subsurface sedimentary environments (Lake Van, Barents Sea, Equatorial Pacific, and Gulf of Mexico) with different predominant electron-acceptors. Hydrogenases constitute a diverse family of enzymes expressed by microorganisms that utilize molecular hydrogen as a metabolic substrate, product, or intermediate. The assay reveals the potential for utilizing molecular hydrogen and allows qualitative detection of microbial activity irrespective of the predominant electron-accepting process. Because the method only requires samples frozen immediately after recovery, the assay can be used for identifying microbial activity in subsurface ecosystems without the need to preserve live material. We measured potential hydrogen oxidation rates in all samples from multiple depths at several sites that collectively span a wide range of environmental conditions and biogeochemical zones. Potential activity normalized to total cell abundance ranges over five orders of magnitude and varies, dependent upon the predominant terminal electron acceptor. Lowest per-cell potential rates characterize the zone of nitrate reduction and highest per-cell potential rates occur in the methanogenic zone. Possible reasons for this relationship to predominant electron acceptor include (i) increasing importance of fermentation in successively deeper biogeochemical zones and (ii) adaptation of H_2_ases to successively higher concentrations of H_2_ in successively deeper zones.

## Introduction

Microorganisms in subsurface environments compete for electron donors and acceptors as they do in most surface environments (D'Hondt et al., 2002). Availability of electron donors and acceptors usually decreases with sediment depth (Froelich et al., 1979; Middelburg, 1989). The predominant terminal electron acceptor is typically inferred to change with sediment depth (e.g., Froelich et al., 1979), with reduction of dissolved O_2_, ${\text{NO}}_{3}^{-}$, Mn^4+^, Fe^3+^, and ${\text{SO}}_{4}^{2-}$ predominating at successively greater depths. After these oxidants are largely exhausted, only fermentation and methanogenesis are left to degrade organic matter (Martens and Berner, 1974; Froelich et al., 1979).

This standard model often provides a good first-order description of the vertical succession of principal terminal electron-accepting activities. However, several studies have shown that multiple organic matter-degrading processes (${\text{SO}}_{4}^{2-}$ reduction, metal reduction, and methanogenesis) co-exist in the same depth intervals of deep subseafloor sediment (e.g., Mitterer et al., 2001; D'Hondt et al., 2002, 2004, 2014; Wang et al., 2008; Holmkvist et al., 2011). Some of these studies show that methanogenesis occurs in zones where sulfate-reduction is the predominant terminal electron acceptor (Mitterer et al., 2001; D'Hondt et al., 2002, 2004, 2014; Wang et al., 2008), while others show that iron reduction occurs deep in the sulfate-reducing zone (Wang et al., 2008; D'Hondt et al., 2014) or that sulfate reduction occurs deep in the “methanogenic” zone (Holmkvist et al., 2011). These deviations from the generally assumed order of electron acceptor utilization represent a significant challenge when trying to quantify microbial activity in subsurface sediment.

These depth-dependent changes in predominant electron acceptor and the overlap of different electron-accepting processes pose challenges for attempts to identify and quantify microbial activity in the sediment column (Wang et al., 2008; Teske, 2012). Currently, there are two general approaches to assess microbial activity across different predominant electron-accepting regimes: (i) separately determine rates of different individual processes and convert them to a common currency (e.g., number of carbon atoms oxidized or electrons transferred; Canfield et al., 1993; D'Hondt et al., 2004) or (ii) measure a parameter that is widely distributed but independent of any specific metabolic process.

The situation is further complicated by the fact that some measurements quantify potential activities (e.g., by measurement of *ex situ* rates) while others quantify actual *in situ* activities. Potential rates of some specific processes, e.g., ${\text{SO}}_{4}^{2-}$ reduction and anaerobic oxidation of methane (AOM) can be quantified with high sensitivity through radiotracer experiments (Kallmeyer et al., 2004; Treude et al., 2005). Potential rates of other processes, such as denitrification, can be quantified using stable isotope tracers (review in Bedard-Haughn et al., 2003).

Several approaches have been made in recent years to develop measures of microbial activity that can be used regardless of terminal electron acceptor (references in Adhikari and Kallmeyer, 2010). Promising approaches include measurements of hydrogenase (H_2_ase) enzyme activity (Schink et al., 1983) and adenosine triphosphate (ATP) concentration (Vuillemin et al., 2013). In a sense, both of these approaches measure potential rates, since they typically rely on measurement of concentration or *ex situ* activity in recovered samples. Soffientino et al. (2006) developed a method for quantifying H_2_ase activity by using a tritium-based H_2_ase assay. This technique has been successfully applied in several studies of subsurface sediment (Soffientino et al., 2006, 2009; Nunoura et al., 2009).

Hydrogen is a key component in anaerobic metabolism and it plays an important role in many biogeochemical reactions (Hoehler et al., 1998; Jørgensen et al., 2001; Nealson et al., 2005). Molecular H_2_ is an important product of fermenters (Laanbroek and Veldkamp, 1982), a byproduct of the nitrogenase reaction by nitrogen-fixing bacteria (Dixon, 1978) as well as a substrate for methanogens (Zeikus, 1977) and sulfate reducers (Jørgensen, 1978b). Several studies have shown that H_2_ is a controlling factor for microbial activity in subsurface environments (Stevens and McKinley, 1995; Anderson et al., 2001; Nealson, 2005; Spear et al., 2005; Hinrichs et al., 2006). Despite the importance of molecular H_2_ in microbial ecosystems, it is rarely quantified in sediment studies, mainly due to technical challenges associated with sampling and sample preservation, measurement of very low concentrations, and potentially high H_2_ turnover rates (Hoehler et al., 2001). In addition to biotic processes, H_2_ can be generated in the subsurface by alteration of young basaltic crust (Stevens and McKinley, 1995; Bach and Edwards, 2003) and by radiolysis of water (Holm and Charlou, 2001; Lin et al., 2005; Blair et al., 2007).

Hydrogenases are intracellular enzymes present in a wide range of microbes (Schink et al., 1983; Vignais and Billoud, 2007). Because hydrogenases facilitate H_2_ utilization and production regardless of terminal electron acceptor, quantification of H_2_ase activity could provide an estimate of microbial activity regardless of predominant terminal electron-acceptor or co-occurrence of different terminal electron acceptors. During the catalytic conversion of H_2_, protons and electrons are produced or consumed based on thermodynamic constraints. Thereby cellular metabolism is facilitated, for example proton gradient formation in the cell membrane to synthesize ATP (Odom and Peck, 1984). Microbes utilize the available electrons from the oxidation of the electron donors to reduce the electron acceptors. The excess energy of this process is harvested through maintaining the proton gradient required for ATP synthesis by which the energy of the catabolic reaction is finally stored in ATP, representing the energy currency in any living cell.

(1) $$\begin{array}{r} {\text{H}}_{2}+\text{E}\Leftrightarrow {\text{E}:\text{H}}_{2}\Leftrightarrow \text{E}+2{\text{H}}^{+}+2{\text{e}}^{-} \end{array}$$

where, E is H_2_ase enzyme and E:H_2_ is the reduced intermediate H_2_ase enzyme complex.

Other enzymes like ATPases and hydrolases are also involved in the pumping of protons; they can also split water molecules to facilitate hydrolytic reactions, however, they are not able to convert molecular H_2_ into protons and electrons. H_2_ase enzymes activate H_2_ molecules by heterolytic cleavage, which is the oxidation of hydrogen to protons (Equation 1). When incubating the sample with tritium, the generated ^3^H^+^ will result in the formation of tritiated water (Equation 2).

(2) $$\begin{array}{l} {}^{1}{\text{H}}_{2}\text{O}+2^{3}{\text{H}}^{+}\Leftrightarrow^{1}{\text{H}}^{+}{+}^{3}{\text{H}}^{+}{+}^{3}{\text{H}}^{1}\text{HO}\Leftrightarrow 2^{1}{\text{H}}^{+}{+}^{3}{\text{H}}_{2}\text{O} \end{array}$$

The overall reaction will be as follows (Equation 3)

(3) $$\begin{array}{l} {}^{3}{\text{H}}_{2}+{\text{H}}_{2}\text{O}\Leftrightarrow^{3}{\text{H}}_{2}\text{O}+{\text{H}}_{2} \end{array}$$

Measurement of the increase of radioactivity of the water can thus be used to infer the rates of hydrogen oxidation catalyzed by the hydrogenase enzymes. According to the modified Michaelis-Menten equation for the reaction shown in Equation (1), by incubating the sample with an excess of molecular H_2_, the forward reaction is strongly favored (Schink et al., 1983) and the backward reaction becomes essentially negligible for the tritium-based assay (Soffientino et al., 2006). Due to the large excess of molecular H_2_, neither its concentration nor the specific activity of ^3^H_2_ (activity of the radioactive substance per mole of total reactant) will significantly change during the course of the incubation, which is a prerequisite for any rate measurement with a radiotracer (Fossing, 1995). The increase in ^3^H_2_O over time yields a rate of ^3^H^+^ generation that is proportional to the total potential activity of all present H_2_ase enzymes. Another factor that strongly favors the forward reaction, i.e., the oxidation of ^3^H_2_ to ^3^H_2_O, is the dilution of the produced ^3^H_2_O in the large H_2_O pool, thereby effectively removing the product.

Different metabolic processes involve different amounts of H_2_ and utilize different types and numbers of H_2_ases with different exchange rates. Moreover, different processes occur simultaneously and their respective contributions to total H_2_ turnover are not known. Consequently, it is essentially impossible to translate hydrogen oxidation into specific metabolic rates. However, it may be possible to identify broad relationships between hydrogen oxidation rates and predominant terminal electron acceptors by comparing H_2_ase activities of sedimentary regimes with different predominant electron acceptors. To test for such relationships, we measured potential H_2_ oxidation in sediment cores from different environments in which different electron acceptors were quantitatively most important. Additionally, we calculated the net turnover rate of the quantitatively most important terminal electron-accepting process. We compared the different datasets to evaluate whether the measured potential hydrogen oxidation rates show any relationship to predominant terminal electron acceptors.

## Materials and sites description

Investigation of different lacustrine and marine sedimentary environments allows testing of the general applicability of the H_2_ase assay in various subsurface environments. We therefore obtained a set of samples that cover a wide range of environments and represent different predominant electron acceptor regimes. We chose the four sampling areas based on sample and data availability, because we needed access to (i) suitable samples for hydrogen oxidation measurements, (ii) porewater chemistry data, and (iii) cell abundance data.

### Lake Van, Turkey

**Figure 1:**
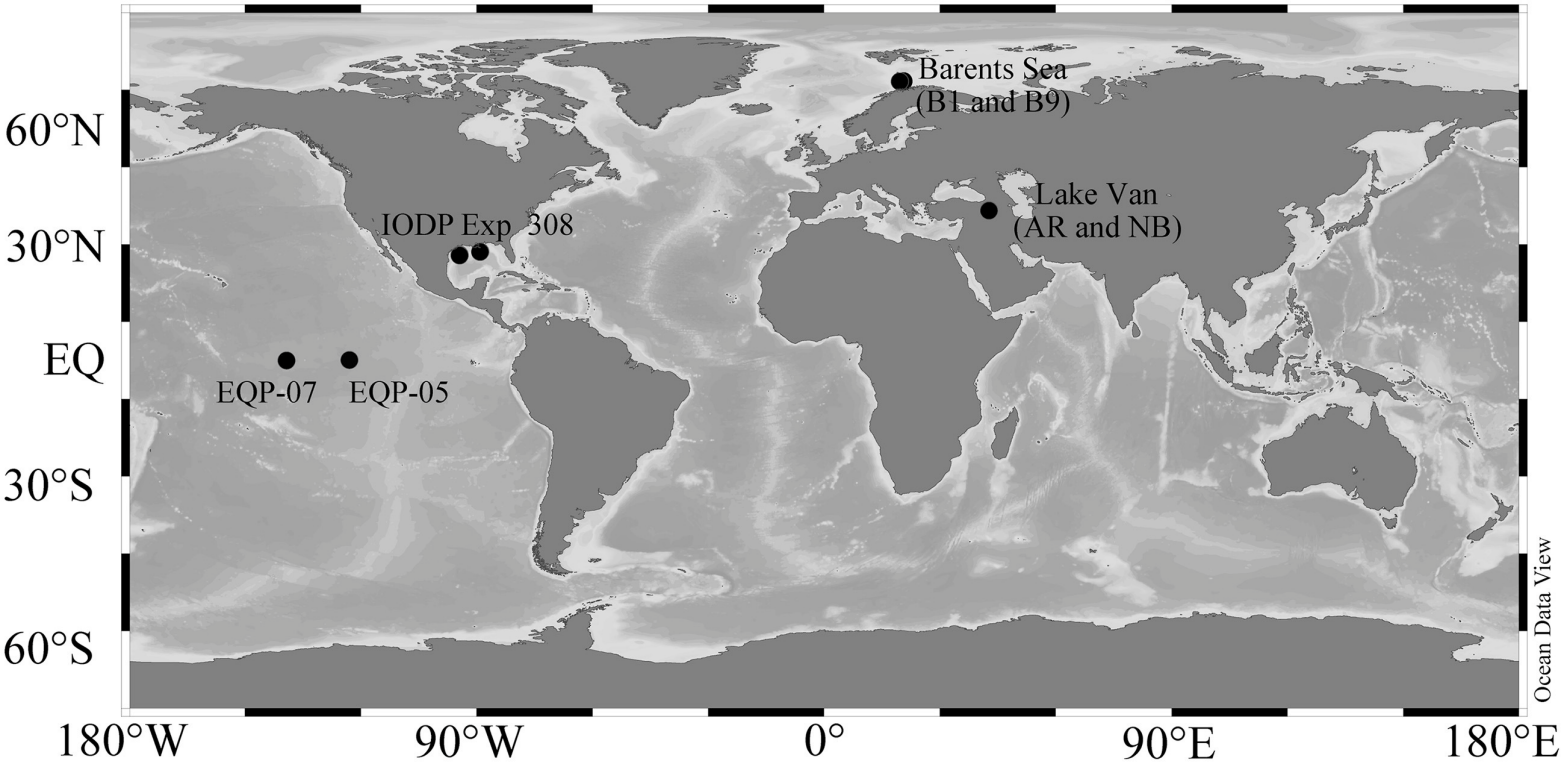
**Study Sites**. The sampling locations Lake Van (AR and NB), Barents Sea (B1 and B9), Equatorial Pacific Sites (EQP-05 and EQP-07), and IODP Expedition 308 Gulf of Mexico (Brazos-Trinity and Ursa Basin) drilling regions plotted in a map using Ocean Data View (http://odv.awi.de/).

Lake Van, one of the largest soda lakes on Earth (Kadioglu et al., 1997), is located in Eastern Anatolia, Turkey. It covers an area of 3570 km^2^ and has a maximum water depth of 460 m (Litt et al., 2009). Bottom water temperature is ca. 3°C (Litt et al., 2012). The lake water has an alkalinity of 155 mEq L^−1^ and a pH of ca. 10, which is mainly due to evaporation and to weathering of nearby volcanic rocks (Kempe et al., 1991). The lake consists of two major basins, which are separated by basement highs and ridges (Figure 1). The Northern Ridge separates the smaller and shallower Northern Basin (NB) from the main Tatvan Basin. Ahlat Ridge (AR) is a small ridge bordering the Ahlat Subbasin, which is located between the NB and the Tatvan Basin. We collected sediment cores during the International Continental Scientific Drilling Program (ICDP) PaleoVan Drilling Operation in summer 2010. For our study, we used short gravity cores of ca. 0.8 m length from the Northern Basin and Ahlat Ridge, taken at water depths of 260 and 375 m, respectively.

### Barents Sea

The Barents Sea is located between the Norwegian Sea to the southwest and the Arctic Ocean to the north. The sampling area is located between the southwestern part of the Loppa High and Ringvassøy-Loppa fault complex (Figure 1). Samples were collected during a 2010 cruise onboard M/S HU Sverdrup II (Nickel et al., 2012). The morphology of the seabed in the sampling area is peculiar as it contains an average of ca. 100 pockmarks per square kilometer. The randomly distributed pockmarks are thought to have been formed by sudden expulsion of fluids or gas from the sediment (Solheim, 1991). The currently inactive pockmarks have a diameter of 10–50 m and an average depth of 1–3 m. Average water depth in the investigated area is around 350 m. Bottom water temperature was around 5°C (Nickel et al., 2012). The samples were retrieved from short gravity cores of ca. 1.5–1.8 m length taken inside the pockmarks at B1 and B9 locations (Nickel et al., 2012).

### Equatorial Pacific

We retrieved samples during the early 2009 R/V Knorr Cruise 195-III in the eastern and equatorial Pacific upwelling area. This region has high primary productivity of ca. 15 mmol C m^−2^ year^−1^ (Røy et al., 2012). Water depths at sites EQP-05 and EQP-07 are 4394 and 4314 m, respectively (Figure 1). Bottom water temperature is approximately 2°C. Using the long core system from the Woods Hole Oceanographic Institution (WHOI), we recovered sediment cores with a maximum length of 35 m.

### Gulf of Mexico

Sediment cores were retrieved during IODP Expedition 308 using an advanced piston corer (APC) and an extended core barrel (XCB). Samples were collected from two sites (U1319 and U1320) at the Brazos-Trinity (BT) Basin and two sites (U1322 and U1324) at the Mars-Ursa Basin (Figure 1) (Flemings et al., 2006). Temperatures of the samples ranged from 4 to 10°C, depending on sediment depth (Long et al., 2008). Volumetric H_2_ase activity and cell abundance data are already published (Nunoura et al., 2009) and we obtained all other geochemical data from the IODP database (http://iodp.tamu.edu/janusweb/general/dbtable.cgi). Porewater extraction, sulfate, and CH_4_ analyses of IODP Expedition 308 samples were carried out on board according to standard IODP protocols (Flemings et al., 2006).

## Methods

### Porewater extraction

Porewater was extracted with an IODP-style titanium/PTFE porewater extraction system (Manheim, 1966) in a hydraulic press (2-column bench top laboratory press, 22 ton max load, Carver Inc., USA). The porewater was filtered through syringe filters (pore size: 0.45 μm) and either analyzed immediately or stored frozen until analysis. During previous expeditions, it was shown that porewater extraction by sediment squeezing generates ${\text{NO}}_{3}^{-}$ concentrations on the scale of tens of micromoles (Schrum et al., 2012). Thus, ${\text{NO}}_{3}^{-}$ concentrations in the porewater samples obtained during the equatorial Pacific expedition were measured in samples obtained using Rhizon soil moisture samplers (Seeberg−Elverfeldt et al., 2005). This practice allowed us to effectively quantify very low ${\text{NO}}_{3}^{-}$ concentrations (<1 μmol L^−1^).

### Porewater analyses

#### Nitrate

During the Equatorial Pacific expedition, ${\text{NO}}_{3}^{-}$ concentrations were analyzed with a Metrohm 844 UV/VIS Compact IC equipped with a 150 × 4.0 mm Metrosep A SUPP 8 150 column. The column temperature was set to 30°C. The eluent was a 10% NaCl solution filtered through a 0.45 μm pore size filter. Approximately 0.8 ml of interstitial water was injected manually into a 250 μL sample loop. UV absorption was determined at 215 nm.

#### Mn and Fe

We analyzed porewater samples for dissolved Mn^4+^ and Fe^3+^ by inductively coupled plasma emission spectrometry (ICP-ES) at Boston University. We acidified the samples at sea with Fisher Optima HNO_3_ and stored them in a refrigerator until analysis. The samples were diluted 10-fold in 2% triple distilled HNO_3_, and calibrated against a seven point calibration curve of matrix matched, spiked International Association for the Physical Sciences of the Ocean standard seawater (IAPSO, *S* = 35, Ocean Scientific International Ltd.) that covered the full concentration range of the unknown samples. We measured Mn^4+^ at 257.609 and 259.373 nm using the ICP-ES monochromator and at 257.61 nm using the ICP-ES polychromator with the Jobin-Yvon Ultima C ICP-ES instrument. Using the ICP-ES monochromator, we measured Fe^3+^ at 238.203 and 259.939 nm, as well as at 259.94 nm using the ICP-ES polychromator. We corrected final concentrations for the 1% acid dilution due to on-board acidification. Analytical precision for both elements is ~3% of the measured value. Accuracy was confirmed by analysis of spiked IAPSO standards that were not included in the original calibration.

#### Sulfate

We quantified dissolved ${\text{SO}}_{4}^{2-}$ in porewater samples from Lake Van and Barents Sea samples using a SYKAM IC system (SYKAM, Fürstenfeldbrück, Germany) equipped with an LCA A14 column, a suppressor (SAMS™, SeQuant, Sweden) and a S3115 conductivity detector. The mobile phase was a 6.25 mmol L^−1^ Na_2_CO_3_ with 0.1 vol% modifier (1g 4-hydroxy-benzonitrile in 50 mL methanol). We eluted the samples at isocratic conditions at 50°C with the eluent flow set to 1 mL min^−1^, injection volume was 30 μL.

During the Equatorial Pacific expedition, we quantified ${\text{SO}}_{4}^{2-}$ concentrations with a Metrohm 861 Advanced Compact IC. The IC was comprised of an 853 CO_2_ suppressor, a thermal conductivity detector, a 150 × 4.0 mm Metrosep A SUPP 5 150 column, and a 20 μL sample loop. A Metrohm 837 IC Eluent/Sample Degasser was coupled to the system. We set the column oven to 32°C. The eluent was 3.2 mmol L^−1^ Na_2_CO_3_, and 1.0 mmol L^−1^ NaHCO_3_. Each sample was analyzed as a 1:50 dilution of interstitial water with 18 MOhm cm^−1^ deionized water. If the samples were anoxic, we added 5 μL of 10% Zn-acetate per mL of analyte to precipitate ZnS.

### Cell enumeration

We took samples for cell enumeration immediately after each core was retrieved. Using cut-off plastic syringes, we placed 2 cm^3^ subsamples in a 15 mL centrifuge tube containing 8 mL of either seawater or NaCl solution with a concentration corresponding to the salinity of the sample. Both solutions were 0.2 μm-filtered and contained 2% formalin as a fixative. We shook the tubes vigorously to create homogenous slurries and stored them at 4°C until analysis.

We undertook cell counts by either (i) direct cell counting or (ii) in cases where cell counts fell below the minimum detection limit of the direct counts, using the cell extraction protocol of Kallmeyer et al. (2008). In cases where cell extraction became necessary, e.g., in deeper sediment layers, we used the two methods with some overlap, to assess the extraction efficiency of the cell separation. In all cases, the two types of counts deviated by less than one standard deviation based on triplicates.

We filtered the sediment slurry or the separated cells onto polycarbonate filters (pore size: 0.2 μm) stained with SYBR Green I according to Morono et al. (2009) and counted the cells using epifluorescence microscopy. Cell counts of samples from IODP Expedition 308 are from Nunoura et al. (2009). For Expedition 308, a 1 mL sediment sample was fixed in 9 mL of 3.7% formaldehyde with PBS and stored in 1:1 (v/v) ethanol:PBS solution. Subsamples were filtered on a polycarbonate membrane, stained with both AO and DAPI, and enumerated by epifluorescence microscopy.

### Modeling of net turnover rates based on porewater concentration profiles

We modeled net turnover rates of predominant terminal electron acceptors using porewater concentration profiles and the approach of Wang et al. (2008). In brief, we smoothed the porewater concentration profiles of predominant terminal electron acceptors with a 5-point Gaussian filter. We used each smoothed concentration profile as an input parameter to model the reaction rate of the profiled chemical by applying the MATLAB® script of Wang et al. (2008). For these calculations, we used a constant formation factor, as well as constant diffusivity and porosity. In addition, we applied a constant sedimentation rate and constant external flow advection velocity near the sediment-water interface. We used a minimum of three concentration data to determine each reaction zone. Modeling of substrate turnover rates from porewater concentration profiles is generally not applicable for near-surface sediment due to disturbance of the sediment-water interface by various factors (e.g., impact of the coring device, bioturbation, etc.; see Jørgensen, 1978a). Thus, the calculated results for the upper meter should be considered carefully. At Lake Van, data from the shallowest samples should still be useful, as anoxic bottom water precludes bioturbation there, and the small coring device that was used probably had little impact on sediment structure (Glombitza et al., 2013). The results of these calculations include positive and negative turnover rates for different depth intervals, with positive rates indicating production of the respective chemical and negative rates indicating consumption. The script of Wang et al. (2008) fixes the modeled concentration at the top and bottom to the uppermost and lowermost measured values. This forcing may cause arbitrary turnover profiles in cases where the measured values show high scatter.

### Hydrogenase enzyme assay

We used a tritium-based H_2_ase assay to measure the potential oxidation rates of hydrogen (Soffientino et al., 2006). We took whole round core pieces or subsamples from the center of each core with cut-off syringes immediately after core retrieval, packed the pieces or subsamples in gas-tight aluminum bags flushed with nitrogen gas, and froze them immediately at −80°C until analysis.

#### Headspace preparation

**Figure 2:**
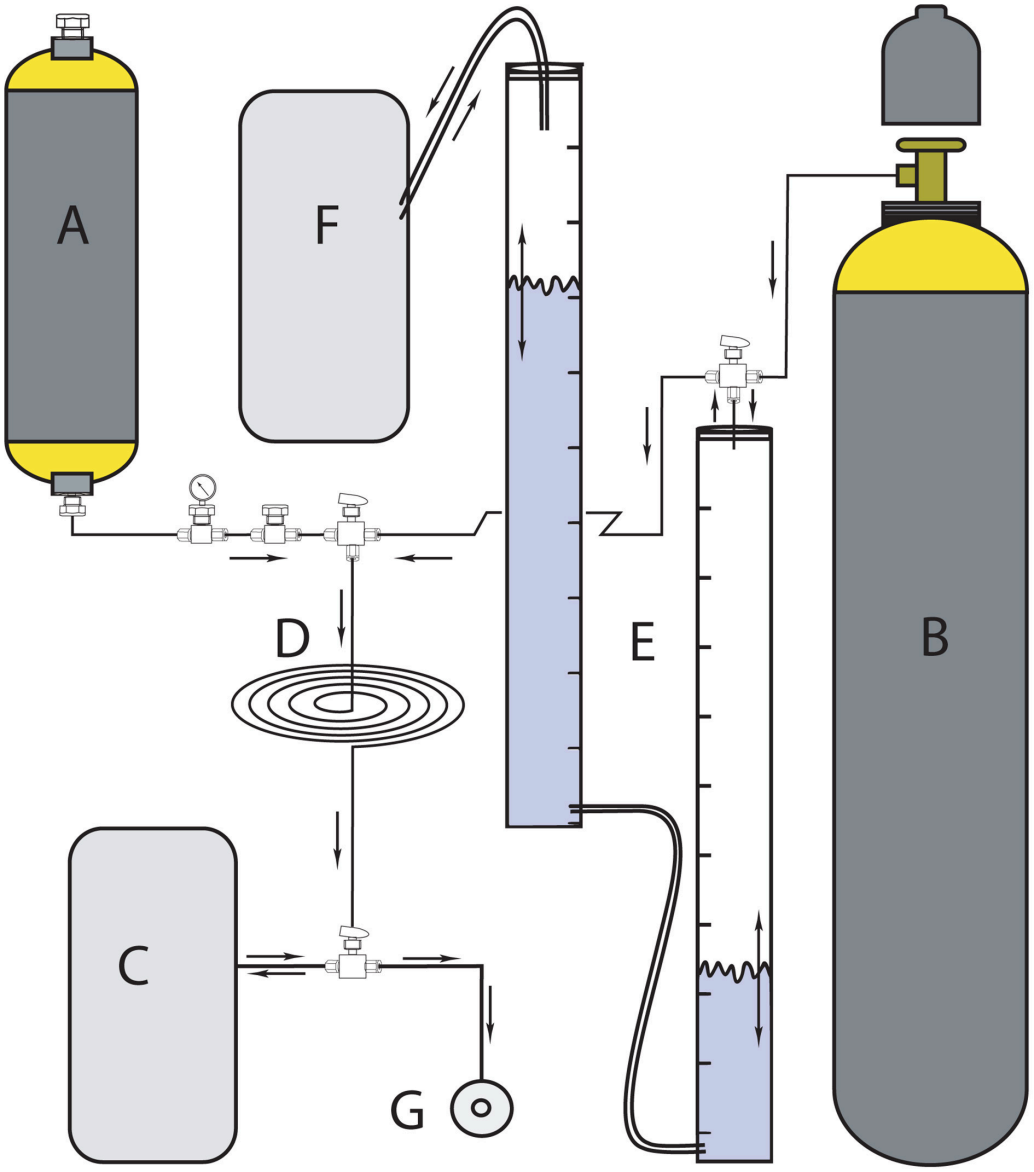
**Headspace ^**3**^H_**2**_/N_**2**_ manifold for storing, diluting, and dispensing ^**3**^H_**2**_ gas to the incubation syringes (modified after Soffientino et al., 2009). (A)** 1-L stainless steel cylinder filled with 37 GBq stock ^3^H_2_ gas; **(B)** cold gas mixture reservoir (20% H_2_ in N_2_); **(C)** secondary headspace reservoir; **(D)** tubing loop; **(E)** two measuring cylinders filled with CrCl_2_ solution to remove traces of O_2_ contained in cold gas mixture before headspace preparation; **(F)** pressure maintaining bag during O_2_ removal, and **(G)** headspace port.

We diluted the carrier-free radioactive ^3^H_2_ (American Radiolabeled Chemicals, Inc.) because its specific activity was too high (37 GBq mL^−1^) to allow for safe and reproducible handling. We prepared the ^3^H_2_ headspace gas by mixing the carrier-free ^3^H_2_ gas with a non-radioactive (20/80% H_2_/N_2_) gas according to Soffientino et al. (2009), considering H_2_ase half-saturation constants and specific activity of ^3^H_2_ (amount of radioactivity per mole of H_2_) to be high enough for sufficient measurement sensitivity when using a slurry to headspace ratio of 1:3. Higher H_2_ concentrations would reduce the specific activity of the ^3^H_2_. To store, dilute and dispense the gas, we constructed a manifold (Figure 2) according to Soffientino et al. (2009) with some minor modifications. In brief, the manifold consists of a 1 L stainless steel cylinder (Figure 2A) for storage of 37 GBq of ^3^H_2_ gas in a mixture of non-radiolabeled 20% H_2_ and 80% N_2_(Figure 2B). The cylinder is connected to the headspace reservoir bag (Figure 2C) via a stainless steel loop with a volume of 11.4 mL (Figure 2D). To prepare the desired specific activity of ^3^H_2_, we diluted one or several volumes of the loop with known volumes of non-radioactive gas. Unlike Soffientino et al. (2009), we added an O_2_ scrubber (Figure 2E) to remove traces of O_2_ from the diluent gas before it is mixed with the ^3^H_2_. The O_2_ scrubber consists of two graduated cylinders filled with chromous chloride (CrCl_2_) solution produced by reacting 1 mol L^−1^ CrCl_3_·6H_2_O in 0.5 mol L^−1^ HCl with Zn granules under a stream of N_2_ (Zhabina and Volkov, 1978). The top of the first cylinder is connected to a non-radiolabeled H_2_/N_2_ gas tank and the stainless steel loop via a three-way valve. Gas entering the cylinder from the tank replaces the CrCl_2_ solution. Through an outlet at the bottom, the gas flows into the second glass cylinder that is connected to a gas-tight aluminum bag (Figure 2F) filled with N_2_ gas to maintain O_2_-free conditions and allow for volume changes. In addition to serve as O_2_ scrubbers, the graduated cylinders were also used to measure the volume of dilution gas. We then flushed the desired volume of non-radioactive gas through the stainless steel loop into the reservoir bag. Finally, we filled the glass syringes with the diluted ^3^H_2_ gas via a sample port (Figure 2G).

We measured specific activity of the headspace gas as described by Soffientino et al. (2009). In brief, we reacted 500-μL ^3^H_2_/N_2_ gas with air in the presence of a heated platinum catalyst to form ^3^H_2_O in a Knallgas-reactor. We trapped the ^3^H_2_O with 5 mL distilled water and quantified radioactivity of the ^3^H_2_O by liquid scintillation counting.

#### Sample preparation

For the measurement of potential hydrogen oxidation rates, we placed ca. 2 g of frozen sediment into a 50 cm^3^ glass syringe barrel fitted with a three-way stopcock and mounted vertically on a manifold. The sediment had to be slurried, so we added 10 mL of sterile anoxic NaCl solution at the concentration necessary to maintain *in situ* salinity in each sample. In order to prevent O_2_ contamination, we performed the process under a continuous stream of N_2_. We processed each sample in triplicates, along with a negative control treated with 0.5 mL saturated HgCl_2_ solution. We incubated all four slurries with 30 cm^3^ headspace gas (^3^H_2_ mixed with non-radioactive 20/80% H_2_/N_2_) on a shaker at 250 rpm at room temperature (ca. 25°C). We collected five subsamples of ca. 0.5 mL at 0.5, 1, 2, 3, and 4 h from each of the slurries and removed unreacted ^3^H_2_ gas by flushing the subsample with N_2_ gas for 10 min and remaining sediment particles by centrifugation. We mixed 100 μL of supernatant with 4 mL Perkin Elmer® Ultima Gold LLT scintillation cocktail for radioactivity quantification by liquid scintillation counting on a Perkin Elmer TriCarb TR2800. The slope of the linear regression over the increase in radioactivity with time is a measure of the potential H_2_ase activity. The killed control should not show any slope and helps to identify abiotic processes that mimic H_2_ase activity (e.g., minerogenic splitting of water molecules).

## Results

**Figure 3:**
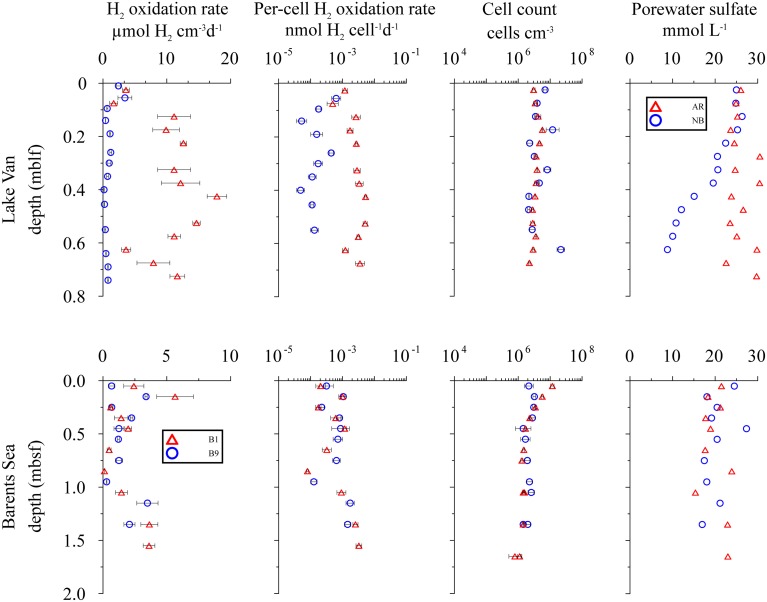
**Distribution of potential hydrogen oxidation rates in sediment samples from Lake Van and Barents Sea**. First column shows sampling depth vs. volumetric potential hydrogen oxidation rates. Second, third, and fourth columns show depth vs. per-cell potential hydrogen oxidation rates, total cell counts and porewater ${\text{SO}}_{4}^{2-}$ concentration, respectively. Error bars represent standard deviation calculated from sample replicates.

**Figure 4:**
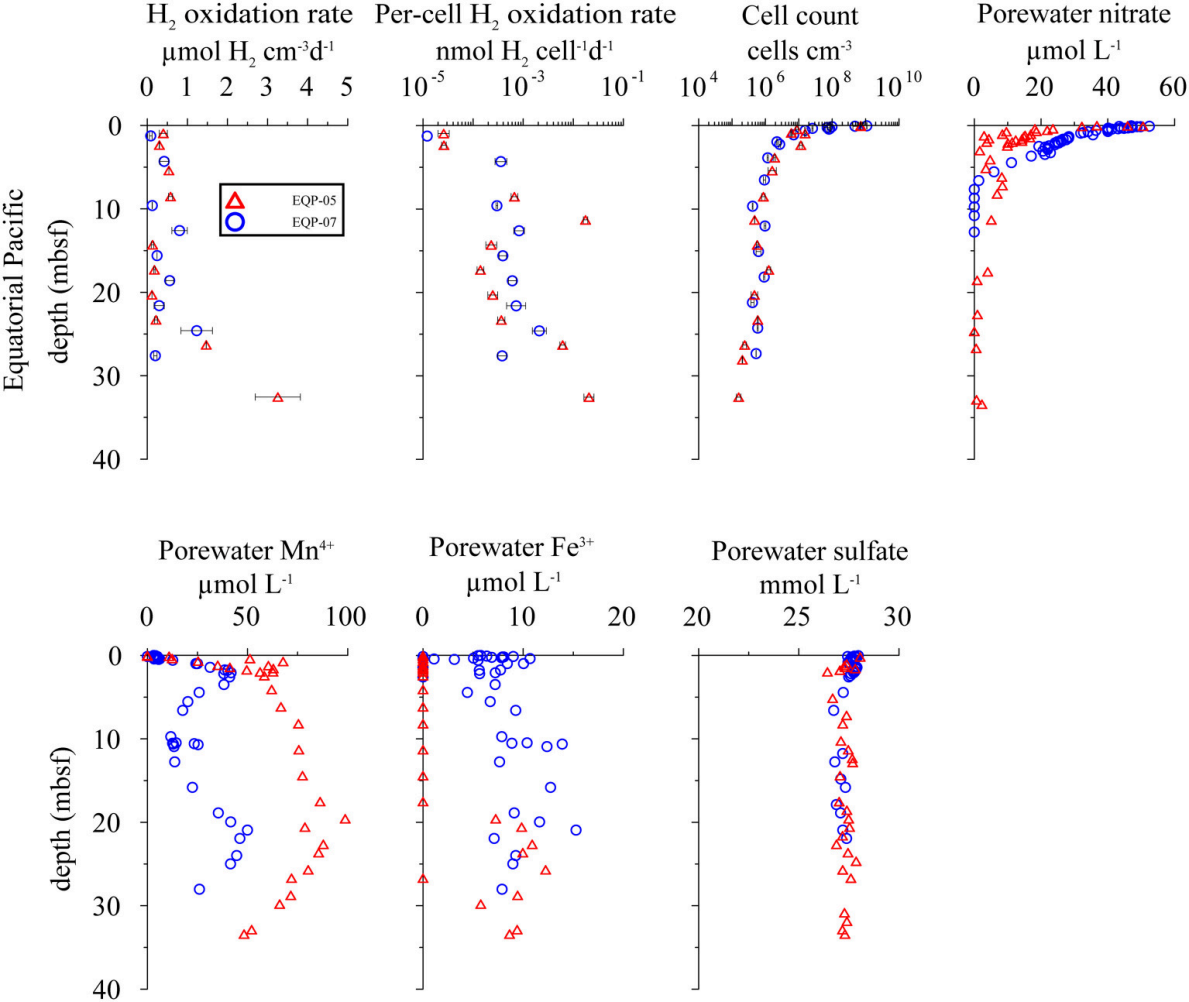
**Distribution of potential hydrogen oxidation rates in sediment samples from Equatorial Pacific Ocean**. EQP-05 (open triangles) and EQP-07 (open circles). From top left to bottom right: volumetric potential hydrogen oxidation rates, per-cell potential hydrogen oxidation rates, total cell counts, porewater ${\text{NO}}_{3}^{-}$, porewater Mn^4+^ and Fe^3+^, and porewater ${\text{SO}}_{4}^{2-}$ concentration. Note that the error bars represent one standard deviation calculated from the measurements from sample replicates.

Using the calculation approach of Soffientino et al. (2006), we quantified potential hydrogen oxidation in all samples from all depths. However, the rates vary significantly between sites and depths. Our killed control experiment shows no positive slope (no ^3^H_2_ incorporation into water) over time. The error bars (Figures 3, 4) represent one standard deviation calculated from the replicates.

### Lake Van

At the Ahlat Ridge (AR) site, we found minimum potential hydrogen oxidation rates of 3.60 ± 1.08 μmol H_2_ cm^−3^ d^−1^ in the uppermost layer [0.03 m below lake floor (mblf)]. Potential rates initially increases with sample depth, reaching a maximum of 17.85 ± 3.07 μmol H_2_ cm^−3^ d^−1^ at a depth of 0.43 mblf, before decreasing again to 11.60 ± 1.98 μmol H_2_ cm^−3^ d^−1^ at the bottom of the core (0.72 mblf). At the Northern Basin (NB) site, potential hydrogen oxidation rates decreases with depth. In the uppermost sediment layer (0.01 mblf), we observed maximum activity of 2.42 ± 0.60 μmol H_2_ cm^−3^ d^−1^. Despite some scattering the potential rates at the NB site decreases by an order of magnitude within a few centimeters and remains in the range of 0.20–1.20 μmol H_2_ cm^−3^ d^−1^ between 0.14 and 0.74 mblf (Figure 3).

At AR, microbial cell numbers are constant around 10^6^ cells cm^−3^ and show little variation with depth. The cell numbers at NB are roughly in the same range but show higher scatter (Figure 3; Kallmeyer et al., 2015). At both sites, pore water ${\text{SO}}_{4}^{2-}$ concentration in the uppermost sediment layer is ca. 26 mmol L^−1^. While at the NB site, concentrations decrease almost linearly to 8 mmol L^−1^ at 0.65 mblf, they remain constant throughout the core, with some scatter at the AR site. In cores from both sites, Glombitza et al. (2013) measured microbial ${\text{SO}}_{4}^{2-}$ reduction rates in radiotracer incubations (^35^${\text{SO}}_{4}^{2-}$). At AR, the radiotracer-based rates are uniformly around 2 nmol cm^−3^ d^−1^, whereas at NB, rates drop from >20 nmol cm^−3^ d^−1^, at the surface to 2 nmol cm^−3^ d^−1^ at 0.2 mblf.

The correlations (*R*^2^) between measured and modeled sulfate concentrations are 0.59 and 0.98 for AR and NB, respectively. The high scatter of the pore water ${\text{SO}}_{4}^{2-}$ data causes the much lower correlation coefficient for AR, although we excluded a few data points because they fall well outside of the general trend and are probably erroneous measurements. Thus, the calculated rates have to be treated with caution (**Figure 6**). Still the calculated *in situ* reaction rates show that sulfate reduction occurs in parts of the sediment cores from both sites, with maximum rates of 2.2 nmol cm^−3^d^−1^ and 4.9 nmol cm^−3^d^−1^ at AR and NB, respectively. For both sites, calculated depth intervals of sulfate production may be an artifact of either high data scatter (AR) or advective exchange of solutes in the uppermost sediment column.

We could not detect nitrate in the porewater. This is not surprising because the bottom water is anoxic (Kaden et al., 2010) and ${\text{NO}}_{3}^{-}$ concentrations are below 1 μmol L^−1^ throughout the water column (Reimer et al., 2009). Methane was below the detection limit of about 0.06 μmol L^−1^ in all samples.

### Barents Sea

Potential hydrogen oxidation rates at both sites in the Barents Sea (B1, B9) vary between 0.12 and 5.65 ± 2.88 μmol H_2_ cm^−3^ d^−1^ (Figure 3). Except for the two uppermost samples, where cell counts of site B1 are higher by up to an order of magnitude, they are almost identical at both sites and vary with depth from 10^6^ to 10^7^ cells cm^−3^. At both sites, ${\text{SO}}_{4}^{2-}$ concentration is around 20 mmol L^−1^ throughout the cores and shows considerable scatter. The ${\text{SO}}_{4}^{2-}$ profiles indicate that microbial ${\text{SO}}_{4}^{2-}$ reduction is very low over the depth interval covered by the cores. We calculated *in situ* net reaction rates from combined sulfate data from both sites. Correlation between measured and smoothed sulfate concentrations is relatively low (*R*^2^ = 0.62). Except for the uppermost 25 cm, where the calculated rate is 4.01 nmol cm^−3^d^−1^ (**Figure 6**), the rates are around zero or even positive, which is in agreement with *in situ* rates measured in close vicinity to our sample sites. (Nickel et al., 2012) found very low sulfate reduction rates of <100 pmol cm^−3^ d^−1^. We could not detect nitrate or CH_4_ in these cores.

### Equatorial Pacific Ocean

At both EQP sites, potential hydrogen oxidation rates are relatively low, with values between 0.08 ± 0.07 and 3.26 ± 1.13 μmol H_2_ cm^−3^ d^−1^. We observed a slight increase in rates in the lowermost samples of EQP-05 (Figure 4). Cell abundance is up to 10^9^ cells cm^−3^ in the uppermost sediment layer (two to three orders of magnitude higher than in the other sediment analyzed for this study). Cell numbers decrease exponentially and remain between 10^5^ and 10^6^ cells cm^−3^ at depths >5 m below sea floor (mbsf) at both EQP sites (Figure 4). Porewater ${\text{SO}}_{4}^{2-}$ concentrations are near seawater values (28 mmol L^−1^) through the entire cored interval. Consequently, the calculated reaction rates are near zero. Only in the uppermost sediment did we observe maximum ${\text{SO}}_{4}^{2-}$ reduction rates of 4 and 109 pmol cm^−3^d^−1^ for EQP-05 and EQP-07, respectively (**Figure 6**). At EQP-05, the correlation between measured and smoothed ${\text{SO}}_{4}^{2-}$ concentrations is very low (*R*^2^ = 0.38), whereas at EQP-07 there is a better fit (*R*^2^ = 0.85).

Nitrate is detectable with maximum concentrations of ca. 50 μmol L^−1^ close to the sediment-water interface. Concentrations decrease steadily to the minimum detection limit of ca. 1 μmol L^−1^ at around 2 and 7 mbsf at Sites EQP-05 and EQP-07, respectively (Figure 4). The strong decrease with depth and the little scatter of the pore water data allowed for good calculation of net nitrate reduction rates at both sites and the smoothed concentration data correlate very well with the measured data (*R*^2^ = 0.86 and 0.99 for EQP-05 and EQP-07, respectively). For both sites, the calculation indicates nitrate production in the uppermost layer, followed by a zone of denitrification below. However, for most of the depth profile the calculated rates are very close to zero (**Figure 6**).

At EQP-05, Mn^2+^ concentration increases steeply over the upper 5 mbsf, from below detection to 67.75 μmol L^−1^. Manganese concentrations further increase with a lower gradient to a maximum of 98.76 μmol L^−1^ at 19.58 mbsf, and then steadily decrease with depth. Over the upper 3–4 mbsf of EQP-07, dissolved Mn^2+^ concentrations are almost identical to those at EQP-05. Below this depth they decrease to 11.73 μmol L^−1^ at 9.73 mbsf before they increase again to a second maximum of 49.97 μmol L^−1^ at 20.93 mbsf. The smoothed concentration profile correlates very well with the measured data for Mn^2+^ at both sites (*R*^2^ = 0.91 and 0.93). For EQP 5 calculated Mn^2+^ production in the uppermost meter is up to 2.5 pmol cm^−3^d^−1^, followed by rates very close to zero at greater depths. For EQP7, the Mn^2+^ concentration profile looks quite different, and consequently also the calculated turnover rates. The strong change from almost −6 to 4 pmol cm^−3^d^−1^ over the upper few centimeters is followed by rates very close to zero, changing back and forth between positive and negative values.

Dissolved Fe^2+^ concentrations show a different trend than Mn^2+^. Dissolved Fe^2+^ concentrations at EQP-05 are below the detection limit until a depth of 17.52 mbsf, and then increase to a maximum of 10.90 μmol L^−1^ at 22.64 mbsf. At EQP-07, dissolved Fe^2+^ concentration varies between 5 and 13 μmol L^−1^ over the entire depth of the cored interval. There is a second maximum concentration peak at approximately the same depth at both sites. The smoothed Fe^2+^ concentration data correlate very well with the measured data at site EQP-05 (*R*^2^ = 0.83), but poorly at EQP-07 (*r*^2^ = 0.52), This poor EQP07 correlation is caused by the high scatter in Fe^2+^ data at EQP-07. Iron reduction rates for EQP-05 and EQP-07 are below 4.7 pmol cm^−3^d^−1^ (**Figure 6**).

### Gulf of Mexico

**Figure 5:**
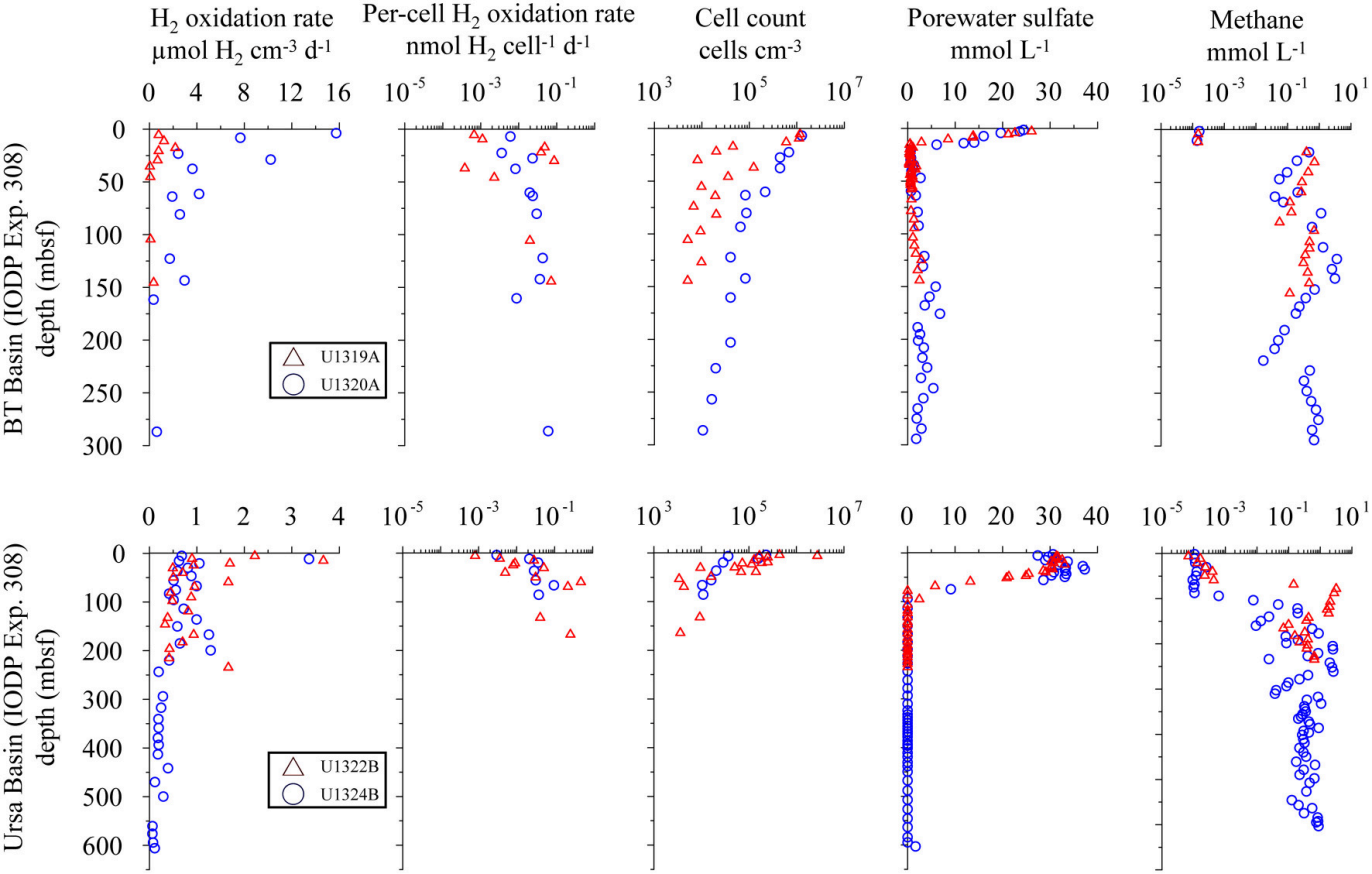
**Distribution of potential hydrogen oxidation rates in sediment samples from IODP expedition 308**. Sites U1319 and U1322 (open triangles) and sites U1320 and 1324 (open circles). From left to right: volumetric potential hydrogen oxidation rates, per-cell potential hydrogen oxidation rates, total cell counts, porewater ${\text{SO}}_{4}^{2-}$ concentration, and headspace CH_4_ gas measurement. Data were taken from IODP database.

At all sites from IODP Expedition 308 (U1319, U1320, U1322, U1324), potential hydrogen oxidation rates are below 5 μmol H_2_ cm^−3^ d^−1^ at all depths, except for three samples from the upper 30 mbsf at site U1320 (Figure 5). These three samples show potential rates of up to 16 μmol H_2_ cm^−3^ d^−1^.

Cell counts are in the range of 10^6^–10^7^ cells cm^−3^ close to the sediment-water interface and decrease to 10^3^–10^4^ cells cm^−3^ at greater depth at all sites. The cell count profiles for sites U1322 and U1324 drop so rapidly below the minimum detection limit around 5^*^10^3^ cells cm^−3^ that there are no cell count data for depth below 150 and 100 mbsf, respectively. Except for Site U1320, cell concentrations at all sites show high scatter, but decline more or less logarithmically with depth.

**Figure 6:**
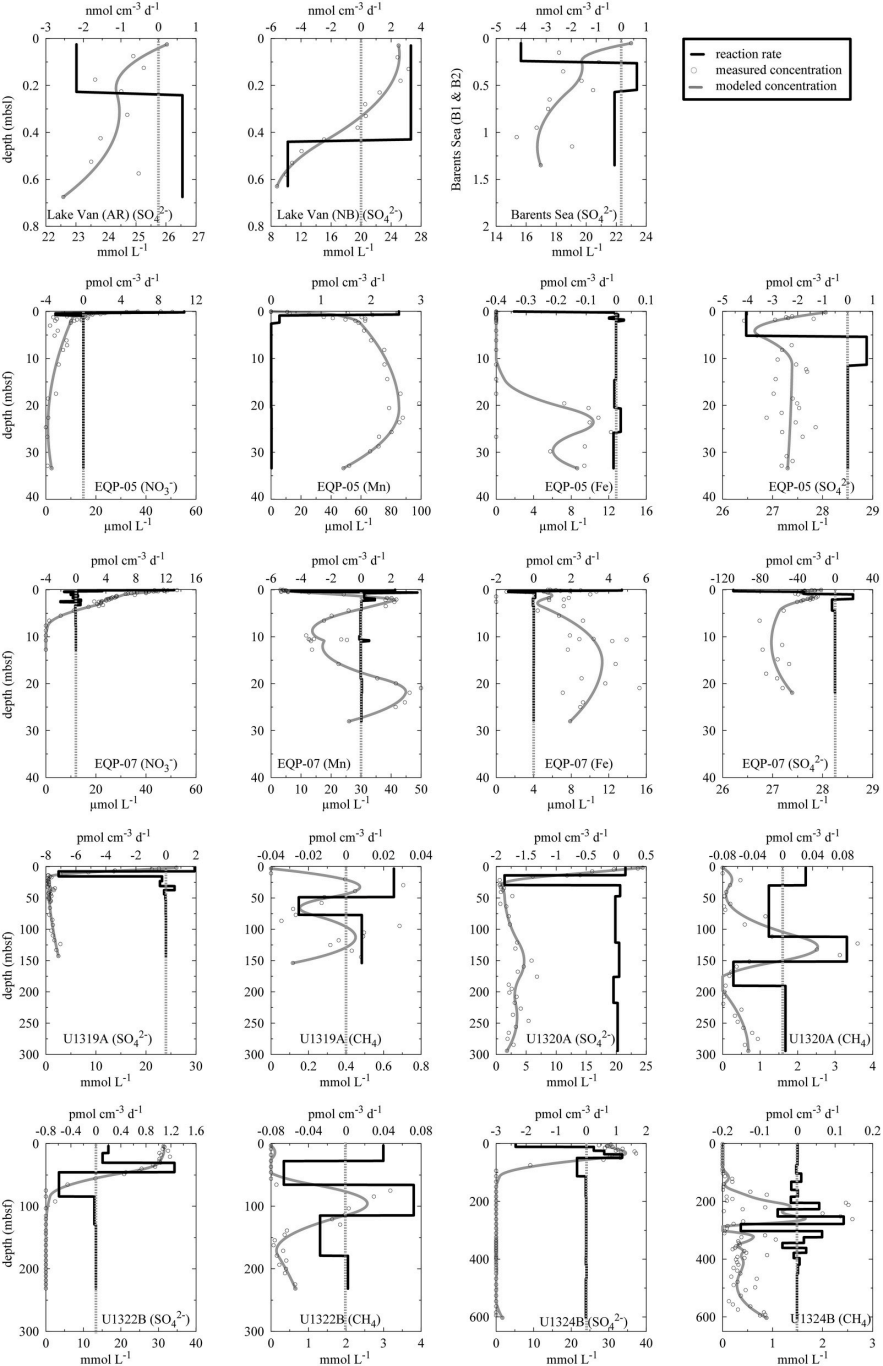
**Calculated reaction rates**. Y-axis refers to depth in meter. Top and bottom x-axes refer to reaction rates and concentrations, respectively. Open circles are measured concentration data, gray solid lines are modeled concentrations, and dark solid lines are modeled reaction rates. Positive values refer to net production and negative values refer to net consumption. Please note different units on the x-axes. Note that the top and bottom values of the smoothed profiles are fixed to the upper- and lower-most measured values.

At Sites U1319 and U1320, dissolved ${\text{SO}}_{4}^{2-}$ is completely depleted within the upper 10 mbsf, whereas at Sites U1322 and U1324, ${\text{SO}}_{4}^{2-}$ concentration remains near seawater concentration in the upper 30 m and 50 m of sediment, respectively before dropping linearly and reaching the detection limit between 80 and 100 mbsf. The ${\text{SO}}_{4}^{2-}$ profile is mirrored by the CH_4_ concentration profile, with values between 0.62 and 6.2 mmol L^−1^ in the sulfate-free zone and low concentrations (<0.6 μmol L^−1^) where ${\text{SO}}_{4}^{2-}$ is present. The sulfate-methane transition zone (SMTZ) can be identified at all four sites, but differs in depth below seafloor and thickness (Figure 5). Measured and smoothed ${\text{SO}}_{4}^{2-}$ concentrations showed a very good correlation (*R*^2^ = 0.99) at all sites (Figure 6). For ${\text{SO}}_{4}^{2-}$, the calculated turnover rates show similar profiles for U1319 and U1320, with a narrow subsurface maximum sulfate reduction zone of −7 and −1.8 pmol cm^−3^d^−1^, respectively, followed by near-zero values for the rest of the core. The calculated ${\text{SO}}_{4}^{2-}$ turnover rates for U1322 and U1324 look considerably different, with more changes between positive and negative rates and a larger positive subsurface peak. The reason for this difference may partly be that the model fixes the concentration at top and bottom to the measured concentration, which may have led to arbitrary results for U1322. For U1324, there seems to be loss of ${\text{SO}}_{4}^{2-}$ to the overlying water as concentrations in the upper 50–60 mbsf are higher than in the overlying water; this is rather unusual for marine sediments.

For CH_4_, we found reasonably good fits of measured concentrations to smoothed concentrations at Sites U1320A (*R*^2^ = 0.83) and U1322B (*R*^2^ = 0.82) and low correlations at Sites U1319A (*R*^2^ = 0.57) and U1324B (*R*^2^ = 0.53). The high scatter in the concentration data from the latter two sites prevents more accurate modeling and the results should be used with great caution. Still, zones of maximum activity can be identified.

## Discussion

### Evaluation of the hydrogen oxidation measurements

Our measurements only delivered potential rates as the conditions under which we performed the incubations differ in several ways from *in situ* conditions. Our use of previously frozen samples, slurries, room temperature, and excess headspace H_2_ all cause deviation from *in situ* conditions.

We used previously frozen samples for all of our hydrogen oxidation measurements. Use of previously frozen material in incubation experiments is unusual for metabolic rate measurements. However, we are measuring potential hydrogen oxidation rates in the sediments, not metabolic activity. Although freezing samples prior to analysis might have damaged cells due to ice crystal formation, cellular integrity is of minor concern for this study; as long as the H_2_ase enzyme remains intact and it finds a redox partner like NAD or ferredoxin, it remains functional. If cells lyse, co-localization of redox partners may be perturbed, which would affect enzymatic rates. Consequently, the enzymes may be less likely to find such a partner in lysed cells than in intact cells. However, extracellular H_2_ases have been reported in soils (Häring et al., 1994), indicating that even extracellular enzymes may find such partners.

Little is known about the survival of H_2_ases outside of cells, as they have not been reported from aquatic or subsurface environments except soil. We therefore assume that all hydrogen oxidation catalyzed by hydrogenase enzymes is related to cells that were physiologically intact at the time of sampling. The samples were immediately frozen in a nitrogen atmosphere and thawed only inside the nitrogen-filled reaction vessel so degradation of biomolecules was minimized. Given the immediate freezing, storage, and thawing under nitrogen and the short incubation time, we assume that degradation of the enzyme is of minor concern, given the orders of magnitude variability of the normalized potential per-cell rates. The results of Soffientino et al. (2009) point toward the same interpretation. They assessed the effects of freezing and thawing on hydrogen oxidation and found 18% higher hydrogen oxidation in fresh sediment than in previously frozen samples, but also noted that more research would help to better constrain the effects of freezing. Due to the availability of only frozen samples, we could not run separate experiments on frozen and fresh material to evaluate this effect.

We made slurries for all of our incubations. Slurrying obviously disturbs sedimentary structure, which might have an effect on the potential turnover rates. Radiotracer ${\text{SO}}_{4}^{2-}$ reduction-rate measurements done in slurries have resulted in higher rates than measurements made with intact sediment (Jørgensen, 1978b). Similar findings have been reported for AOM experiments (Treude et al., 2003, 2005). We therefore suspect that slurrying may have a similarly positive effect on our measured potential hydrogen oxidation rates.

We carried out our incubations at room temperature (ca. 25°C), which is considerably higher than *in situ* temperatures. Several studies have shown that increasing temperature has a positive effect on metabolic rates (Arnosti et al., 1998; Sagemann et al., 1998; Thamdrup and Fleischer, 1998; Finke and Jørgensen, 2008), usually leading to an increase by a factor of 3–5. One of the few studies that measured the temperature dependence of enzyme activity in sediments also found a ca. three to five-fold increase in polysaccharide hydrolysis between 5 and 25°C (Arnosti et al., 1998). Given the order-of-magnitude approach of our study we consider these changes to be negligible. Moreover, all samples have a relatively narrow *in situ* temperature range between 2 and 10°C, so the temperature effect should be similar in all samples.

Our most important deviation from *in situ* conditions is incubation of our sediment slurries with excess H_2_ in the headspace (20%), leading to complete saturation of the samples with H_2_ (ca. 16 μmol L^−1^), which is controlled by its solubility. In natural sedimentary systems, H_2_ concentrations can be two to three orders of magnitude lower than in our incubations (e.g., Hoehler et al., 1998). Hoehler et al. (2002) showed that microorganisms can maintain H_2_ concentrations at a thermodynamically favorable level, for example, ${\text{SO}}_{4}^{2-}$ reducers maintain H_2_ concentration at such a low level that conditions can become thermodynamically unfavorable for CO_2_ reducers. By supplying excess H_2_, our experiments over-ride these naturally low concentrations and allow for unlimited H_2_ oxidation with negligible back-reaction according to Michaelis-Menten kinetics (Soffientino et al., 2009; Equation 3). In short, our measurements provide rates of H_2_ oxidation that are facilitated by H_2_ases regardless of the *in situ* direction of the process. Because fermenters produce most of the H_2_ that is used by other microbes, about half of the measured potential hydrogen oxidation should account for H_2_ production under *in situ* conditions.

The excess of H_2_ is necessary to ensure comparable conditions in all samples, because we do not have any information about *in situ* concentrations. Experiments under *in situ* temperature and H_2_ partial pressure might provide results closer to *in situ* rates but incubating samples under high hydrostatic pressure and maintaining precise H_2_ partial pressure entail additional technical challenges (Sauer et al., 2012) that are beyond the scope of this study. As shown by Soffientino et al. (2006), saturating the slurry with H_2_ and constant shaking during the incubation is necessary to achieve a linear increase in the concentration of ^3^H_2_O (i.e., constant enzyme activity) because the reaction is diffusion-limited. The low solubility of H_2_ in water (ca. 16 μmol L^−1^ at 25°C) is partially countered by its high diffusivity (8.69 × 10^−9^ m^2^ s^−2^ at 25°C; Jørgensen, 2000, which is similar to the diffusivity of water ca. 2.5 × 10^−9^ m^2^ s^−2^ at 25°C), leading to a diffusion time of <0.1 ms for a distance of 1 μm. This distance is larger than the diameter of an average microbial cell in subsurface sediment (Kallmeyer et al., 2012). Although intracellular membranes restrict diffusion to some degree, we think that it is safe to assume that all intracellular reactants are in equilibrium with the surrounding water over the time course of the experiment.

Volumetric potential hydrogen oxidation (H_2_ turnover per volume sediment) varies by about two orders of magnitude between sites and depths, without any visible correlation with depth, sites, or biogeochemical zones. We interpret this as indicating that H_2_ases are ubiquitously distributed and a potentially active microbial community is present at all sampled depths at all sites in this study (Figures 3–5). Except for disruption of the cells due to freezing and the consequent potential loss of a redox partner (Soffientino et al., 2009), the deviations of our incubations from *in situ* conditions point toward our measured rates being higher than *in situ* rates. Because every sample likely contains a mixture of different H_2_ases with different substrate specificities and exchange rates, it seems unlikely that activity will increase linearly with substrate concentration over several orders of magnitude. Still, it might be safe to assume that our measured rates overestimate *in situ* turnover by at least an order of magnitude, given the higher temperature, slurrying, and saturation with H_2_. With decreasing redox potential, H_2_ plays an increasingly important role in electron-acceptor processes, so the level of overestimation may differ between redox zones. Nevertheless, the hydrogen oxidation rate measurements indicate potential for microbial activity regardless of the specific catabolic reaction (e.g., metal, ${\text{SO}}_{4}^{2-}$, or CO_2_ reduction), and thus provide the opportunity to detect potential activity throughout the whole sediment column by a single method.

The ability to detect hydrogen oxidation and thus active microbial metabolism by using frozen samples is an advantage over methods that detect activity by measuring a metabolic rate, which requires metabolically active cells. Such methods usually require fresh, or rather non-frozen, sample material. Because the hydrogen oxidation measurement only targets the intact H_2_ase enzyme, microbial cells do not have to be kept alive until analysis. The only requirement is that the samples be frozen immediately after retrieval and kept under anoxic conditions. H_2_ase-catalyzed hydrogen oxidation can thus be detected even after long periods of sample storage. This provides the opportunity to detect microbial activity in sediment retrieved during sampling campaigns where logistical or operational limitations do not allow immediate experimental treatment.

We used porewater chemical concentration profiles and the procedure of Wang et al. (2008) to calculate net turnover rates for relevant substrates at the individual sites. For each site, we separately calculated the turnover rate of the relevant metabolic compound (Figure 6). Perhaps unsurprisingly, we observe no significant correlation between potential volumetric hydrogen oxidation rates and calculated metabolic rates. There are several possible reasons for this lack of correlation. The calculated rates represent only net rates of production or consumption of single compounds, whereas our potential H_2_ oxidation rate measurements provide a qualitative measure of the activity of the entire microbial community. As described by Wang et al. (2008) several metabolic activities can co-occur at the same depth, so any single calculation may fail to account for metabolic diversity. Although the porewater data from the Equatorial Pacific (EQP-05 and EQP-07) allowed calculation of turnover rates for several potentially co-existing metabolic processes, we found no correlation between the ensemble of metabolic rates and measured potential volumetric hydrogen oxidation rates. Due to this absence of correlation between calculated net turnover rates and measured H_2_ oxidation rates, only qualitative assumptions about microbial activity can be made.

Although potential volumetric hydrogen oxidation rates cannot be translated directly into specific turnover rates, per-cell potential hydrogen oxidation rates can be compared within biogeochemical zones and across different zones.

### Hydrogen utilization potential and biogeochemical zonation

Because the volumetric potential hydrogen oxidation rate remains relatively constant with depth despite order-of-magnitude changes in cell abundance, we normalized potential hydrogen oxidation rate by dividing volumetric rates by the number of counted microbial cells (Figures 3–5). The counts include all cells that contain double-stranded DNA, irrespective of metabolic status or capability.

A recent study of Peters et al. (2015) estimated that only about 35% of all microorganisms encode for H_2_ases, suggesting that H_2_ases are not as ubiquitously distributed as previously thought (Soffientino et al., 2006). However, the findings of Peters et al. (2015) may not be fully applicable to subsurface environments as their study covered only 167 (5.7%) archaeal genomes. Archaea and bacteria have recently been reported to occur in roughly equal proportions in subseafloor sediment (Lloyd et al., 2013; Xie et al., 2013). Also, Fe-only H_2_ases were not considered by Peters et al. (2015) but are found in many methanogenic archaea (Lyon et al., 2004) that comprise a significant part of lacustrine and marine subsurface microbial communities (Lipp et al., 2008). In short, methanogenic and methanotrophic archaea are important players in subsurface ecosystems, and the data set of Peters et al. (2015) may not be representative of such environments. Rather, in the samples used in our study, the fraction of microbes that contain H_2_ases is likely >35%. The fact that 93 (55%) of the 167 archaeal genomes covered by the study of Peters et al. (2015) contains [FeFe]- and [NiFe]-H_2_ase homologs suggests that a higher percentage of archaea contain H_2_ases. Even the most negative assumption, that only one third of all microbial cells contained H_2_ases, would introduce a bias of a factor of three. This factor is dwarfed by the variation that we observe per-cell potential hydrogen oxidation rates, which range over four orders of magnitude from site to site (2 × 10^−5^ to 2 × 10^−1^ nmol H_2_ cell^−1^ d^−1^; Figures 3–5). Given this variation in per-cell potential hydrogen oxidation rates, a potential factor-of-three bias in the assumed fraction of cells that encodes for H_2_ases is insignificant.

**Figure 7:**
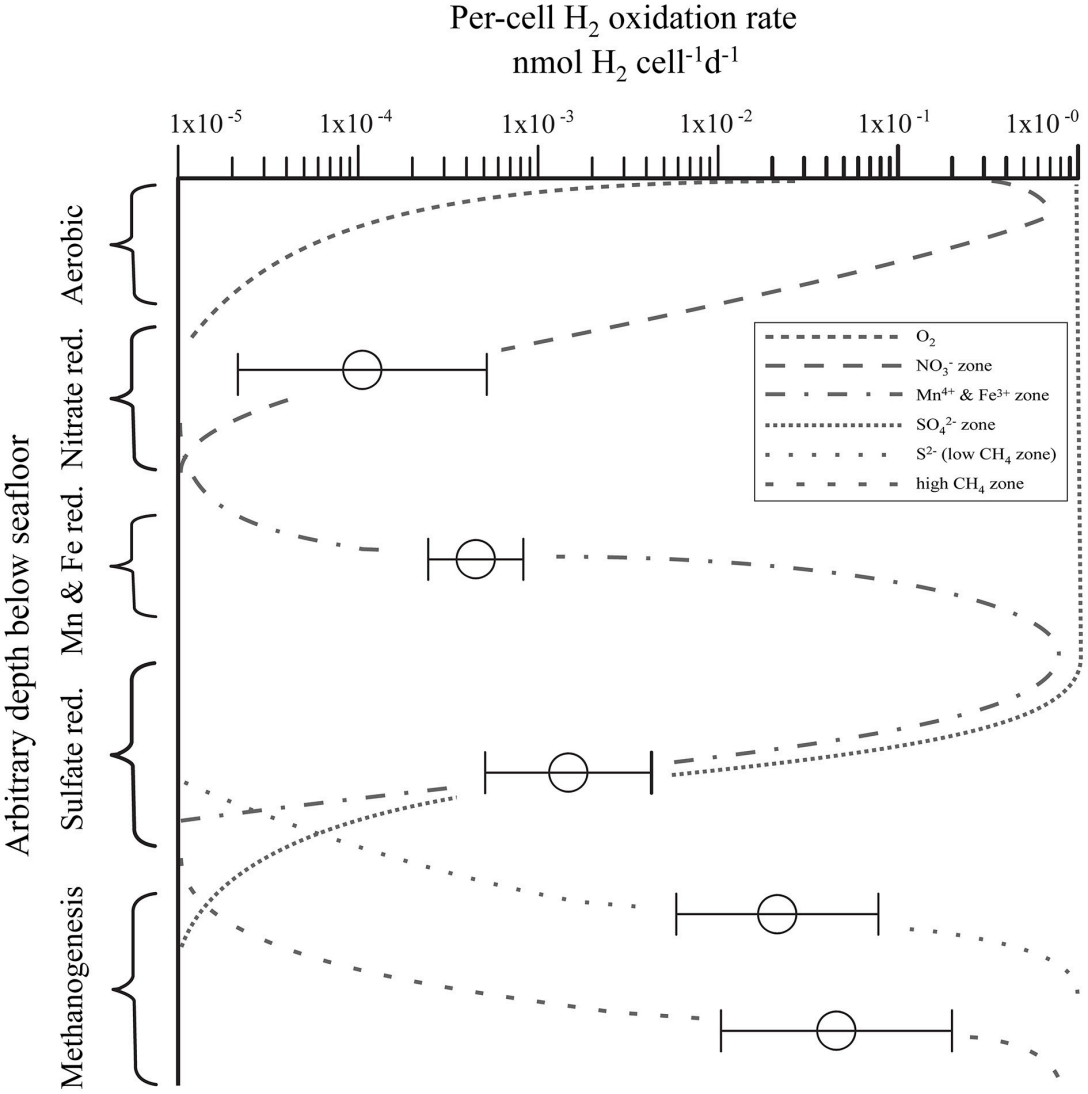
**Relationship of per-cell potential hydrogen oxidation rates to predominant terminal electron acceptor in a set of 93 samples from a subsurface depth range of 0–286 m [Equatorial Pacific, ***N*** = 14; Barents Sea, ***N*** = 20; Gulf of Mexico, ***N*** = 37; Lake Van, Turkey, ***N*** = 22; layout adapted after modification from Megonigal et al. (2005)]**. Vertical zones indicate predominant terminal acceptor. Horizontal axis is per-cell potential hydrogen oxidation rates. Horizontal bars represent standard deviations calculated from multiple sample measurements at different depths and sites but from the same biogeochemical zone.

We selected our sample set to the predominant electron-acceptor zones that typically occur in anoxic sediment (${\text{NO}}_{3}^{-}$ reduction, Mn^4+^ and Fe^3+^ reduction, ${\text{SO}}_{4}^{2-}$ reduction, and methanogenesis zones). To identify common patterns in H_2_ utilization, we grouped the samples into biogeochemical zones based on porewater composition. The first group contains all samples with detectable ${\text{NO}}_{3}^{-}$ (${\text{NO}}_{3}^{-}$ zone). The second group contains samples where metal reduction predominates (Mn and Fe zone). The third group is defined by the presence of ${\text{SO}}_{4}^{2-}$ and the absence of CH_4_ (${\text{SO}}_{4}^{2-}$ zone). We subdivided samples with detectable CH_4_ into a low-CH_4_ (<0.6 μmol L^−1^) zone and a high-CH_4_ (>0.6 μmol L^−1^) zone to separate samples with persistent but very low CH_4_ even in the presence of dissolved ${\text{SO}}_{4}^{2-}$ (e.g., IODP Exp. 308, BT Basin) from those with much higher CH_4_ concentration and usually no ${\text{SO}}_{4}^{2-}$. When plotting the per-cell potential hydrogen oxidation rate relative to the biogeochemical zones (Figure 7) the relationship between per-cell potential hydrogen oxidation activity and predominant metabolic process becomes obvious. Despite some scatter, per-cell potential hydrogen oxidation rates increase in the following order: ${\text{NO}}_{3}^{-}$ zone < Mn^4+^ and Fe^3+^ zone < ${\text{SO}}_{4}^{2-}$ zone < low CH_4_ zone < high CH_4_ zone. The lowest per-cell potential hydrogen oxidation rates (1.2 × 10^−5^−5 × 10^−4^ nmol H_2_ cell^−1^ d^−1^) occur in the ${\text{NO}}_{3}^{-}$ zone of site EQP-05 and site EQP-07. The metal reduction zone exhibits mean per-cell potential hydrogen oxidation rates that lie between those found in the ${\text{NO}}_{3}^{-}$ and ${\text{SO}}_{4}^{2-}$ reduction zones. Per-cell potential hydrogen oxidation rates in the ${\text{SO}}_{4}^{2-}$ zone samples from Lake Van, Barents Sea, and the Equatorial Pacific mostly range between 5 × 10^−4^ and 4 × 10^−3^ nmol H_2_ cell^−1^ d^−1^. In the low CH_4_ zone (<0.6 μmol L^−1^), they fall in the range of 5 × 10^−3^ to 8 × 10^−2^ nmol H_2_ cell^−1^ d^−1^. In the high CH_4_ zone (>0.6 μmol L^−1^), rates are highest with values between 1 × 10^−2^ and 2 × 10^−1^ nmol H_2_ cell^−1^ d^−1^.

There are multiple possible cause(s) of these differences in per-cell potential hydrogen oxidation rates between different redox (or predominant electron-acceptor) zones. They may be due to differences in H_2_ase enzyme kinetics or they may be related to *in situ* H_2_ concentrations. Thus, in the less reducing zones, H_2_ases need to be adapted to very low H_2_ concentration resulting in lower k_m_ (Michaelis constant) values which often means lower V_max_ (maximum turnover rates), whereas in deeper layers, in which fermenters make up a higher proportion of the microbial community, H_2_ concentration may be higher and the enzymes have a higher k_m_ and a higher V_max_. As we carried out our incubations at uniformly high H_2_ concentrations, H_2_ases from deeper zones might be better adapted and work at a higher turnover rate.

With increasing sediment depth (or more precisely, age), organic matter becomes more recalcitrant. Although breakdown of macromolecular organic matter into biologically available monomers continues with increasing burial depth, the amount, and diversity of the bioavailable material decreases. Even highly complex organic molecules can be degraded by nitrate, metal, and sulfate reducers (Widdel and Rabus, 2001; Foght, 2008). In general, the substrate spectrum of microbes becomes smaller with decreasing redox potential, with methanogens having the smallest spectrum and reliant on fermenters to produce the narrow range of compounds (including H_2_) that they can utilize. Thus, with increasingly reducing conditions, the fraction of organic matter that is fermented also increases, which is also concomitant to a greater production of H_2_.

## Conclusions

We measured potential hydrogen oxidation in all samples from multiple depths at several sites that collectively span a wide range of environmental conditions and biogeochemical zones. The ubiquity of this potential activity is consistent with ubiquity of H_2_ utilization in subsurface marine and lacustrine sedimentary environments. Per-cell potential rates increases with increasing sediment depth, from one subsurface redox zone to another.

The tritium-based assay provides information about the microbial community's potential to utilize molecular H_2_. It does not provide actual (*in situ*) rates, because the incubation conditions deviate in many ways from *in situ* conditions. Most obviously, the experimental setup forces all H_2_ases to proceed in the net direction of splitting molecular H_2_ to proton and electrons, irrespective of their role *in situ*. For example, H_2_ases utilized by fermenters, which produce most of the H_2_ in subsurface systems, also contribute to the measured H_2_ oxidation rates, just like H_2_ases that are used *in situ* for H_2_ turnover by terminal oxidizers.

Nonetheless, this assay has potentially significant advantages. It can be used to detect potential microbial activity in subsurface sedimentary environments, regardless of predominant terminal electron-accepting process. And it can be applied to previously frozen sediment, which cannot usually be used for direct measurements of metabolic rates.

Per-cell potential hydrogen oxidation rates appear to vary with predominant electron acceptor at our sites. It is lowest in sediment where ${\text{NO}}_{3}^{-}$ reduction predominates, and successively higher where metal (Mn^4+^ and Fe^3+^) reduction, ${\text{SO}}_{4}^{2-}$ reduction, and CH_4_ production (CO_2_ reduction) predominates. Possible reasons for this relationship to predominant electron acceptor include (i) increasing importance of fermentation in successively deeper biogeochemical zones and (ii) adaptation of H_2_ases to successively higher concentrations of H_2_ in successively deeper zones.

## Author contributions

RA and JK designed the study; RA, JK, JN, CG collected the samples; RA, JN, CA, AD, CG performed the laboratory work; RA, RM, AS, SD, CG, and JK interpreted data; RA wrote the paper; CG, SD, and JK edited the paper; all authors reviewed the manuscript.

### Conflict of interest statement

The authors declare that the research was conducted in the absence of any commercial or financial relationships that could be construed as a potential conflict of interest.

## References

[B1] Adhikari R. R. Kallmeyer J. (2010). Detection and quantification of microbial activity in the subsurface. Chem. Erde-Geochem. 70, 135–143. 10.1016/j.chemer.2010.05.003

[B2] Anderson R. T. Chapelle F. H. Lovley D. R. (2001). Comment on “Abiotic controls on H_2_ production from basalt-water reactions and implications for aquifer biogeochemistry”. Environ. Sci. Technol. 35, 1553–1557. 10.1021/es0015996 11348102

[B3] Arnosti C. Jørgensen B. B. Sagemann J. Thamdrup B. (1998). Temperature dependence of microbial degradation of organic matter in marine sediments: polysaccharide hydrolysis, oxygen consumption, and sulfate reduction. Mar. Ecol. Prog. Ser. 165, 59–70. 10.3354/meps165059

[B4] Bach W. Edwards K. J. (2003). Iron and sulfide oxidation within the basaltic ocean crust: implications for chemolithoautotrophic microbial biomass production. Geochim. Cosmochim. Acta 67, 3871–3887. 10.1016/S0016-7037(03)00304-1

[B5] Bedard-Haughn A. van Groenigen J. W. van Kessel C. (2003). Tracing ^15^N through landscapes: potential uses and precautions. J. Hydrol. 272, 175–190. 10.1016/S0022-1694(02)00263-9

[B6] Blair C. C. D'Hondt S. Spivack A. J. Kingsley R. H. (2007). Radiolytic hydrogen and microbial respiration in subsurface sediments. Astrobiology 7, 951–970. 10.1089/ast.2007.0150 18163872

[B7] Canfield D. E. Jørgensen B. B. Fossing H. Glud R. Gundersen J. Ramsing N. B.. (1993). Pathways of organic carbon oxidation in three continental margin sediments. Mar. Geol. 113, 27–40. 10.1016/0025-3227(93)90147-N 11539842

[B8] D'Hondt S. Jørgensen B. B. Miller D. J. Batzke A. Blake R. Cragg B. A.. (2004). Distributions of microbial activities in deep subseafloor sediments. Science 306, 2216–2221. 10.1126/science.1101155 15618510

[B9] D'Hondt S. Rutherford S. Spivack A. J. (2002). Metabolic activity of subsurface life in deep-sea sediments. Science 295, 2067–2070. 10.1126/science.1064878 11896277

[B10] D'Hondt S. Wang G. Spivack A. J. (2014). The underground economy (energetic constraints of subseafloor life), in Earth and Life Processes Discovered from Subseafloor Environments: A Decade of Science Achieved by the Integrated Ocean Drilling Program (IODP), Vol. 7, eds Stein R. Blackman D. K. Inagaki F. Larsen H-H. (Amsterdam: Elsevier), 127.

[B11] Dixon R. (1978). Nitrogenase - Hydrogenase interrelationships in Rhizobia. Biochimie 60, 233–236. 10.1016/S0300-9084(78)80819-0 667179

[B12] Finke N. Jørgensen B. B. (2008). Response of fermentation and sulfate reduction to experimental temperature changes in temperate and Arctic marine sediments. ISME J. 2, 815–829. 10.1038/ismej.2008.20 18309360

[B13] Flemings P. Behrmann J. John C. Scientists (2006). Expedition reports Gulf of Mexico hydrogeology, in Proceedings of the IODP 308 (College Station, TX: IODP).

[B14] Foght J. (2008). Anaerobic biodegradation of aromatic hydrocarbons: pathways and prospects. J. Mol. Microbiol. Biotechnol. 15, 93–120. 10.1159/000121324 18685265

[B15] Fossing H. (1995). 35s-radiolabeling to probe biogeochemical cycling of sulfur, in Geochemical Transformations of Sedimentary Sulfur, Vol. 612, eds Vairavamurthy M. A. Schoonen M. A. A. (Washington, DC: American Chemical Society), 348–364.

[B16] Froelich P. N. Klinkhammer G. P. Bender M. L. Luedtke N. A. Heath G. R. Cullen D. (1979). Early oxidation of organic-matter in pelagic sediments of the eastern equatorial atlantic - suboxic diagenesis. Geochim. Cosmochim. Acta 43, 1075–1090. 10.1016/0016-7037(79)90095-4

[B17] Glombitza C. Stockhecke M. Schubert C. J. Vetter A. Kallmeyer J. (2013). Sulfate reduction controlled by organic matter availability in deep sediment cores from the saline, alkaline Lake Van (Eastern Anatolia, Turkey). Front. Microbiol. 4:209. 10.3389/fmicb.2013.00209 23908647 PMC3725400

[B18] Häring V. Klüber H. Conrad R. (1994). Localization of atmospheric H2-oxidizing soil hydrogenases in different particle fractions of soil. Biol. Fertil. Soils 18, 109–114. 10.1007/BF00336455

[B19] Hinrichs K. U. Hayes J. M. Bach W. Spivack A. J. Hmelo L. R. Holm N. G.. (2006). Biological formation of ethane and propane in the deep marine subsurface. Proc. Natl. Acad. Sci. U.S.A. 103, 14684–14689. 10.1073/pnas.0606535103 16990430 PMC1595412

[B20] Hoehler T. M. Albert D. B. Alperin M. J. Bebout B. M. Martens C. S. Des Marais D. J. (2002). Comparative ecology of H_2_ cycling in sedimentary and phototrophic ecosystems. Antonie van Leeuwenhoek 81, 575–585. 10.1023/A:1020517924466 12448753

[B21] Hoehler T. M. Alperin M. J. Albert D. B. Martens C. S. (1998). Thermodynamic control on hydrogen concentrations in an anoxic sediment. Geochim. Cosmochim. Acta 62, 1745–1756. 10.1016/S0016-7037(98)00106-9

[B22] Hoehler T. M. Alperin M. J. Albert D. B. Martens C. S. (2001). Apparent minimum free energy requirements for methanogenic Archaea and sulfate-reducing bacteria in an anoxic marine sediment. FEMS Microbiol. Ecol. 38, 33–41. 10.1111/j.1574-6941.2001.tb00879.x

[B23] Holm N. G. Charlou J. L. (2001). Initial indications of abiotic formation of hydrocarbons in the rainbow ultramafic hydrothermal system, Mid-Atlantic Ridge. Earth Planet. Sci. Lett. 191, 1–8. 10.1016/S0012-821X(01)00397-1

[B24] Holmkvist L. Ferdelman T. G. Jørgensen B. B. (2011). A cryptic sulfur cycle driven by iron in the methane zone of marine sediment (Aarhus Bay, Denmark). Geochim. Cosmochim. Acta 75, 3581–3599. 10.1016/j.gca.2011.03.033

[B25] Jørgensen B. (1978a). A comparison of methods for the quantification of bacterial sulfate reduction in coastal marine sediments: II. Calculation from mathematical models. Geomicrobiol. J. 1, 29–47. 10.1080/01490457809377722

[B26] Jørgensen B. B. (1978b). A comparison of methods for the quantification of bacterial sulfate reduction in coastal marine sediments 1. Measurement with radiotracer techniques. Geomicrobiol. J. 1, 11–27. 10.1080/01490457809377721

[B27] Jørgensen B. B. (2000). Bacteria and marine biogeochemistry, in Marine Geochemistry, eds Schulz H. Zabel M. (Berlin: Springer), 173–207.

[B28] Jørgensen B. B. Weber A. Zopfi J. (2001). Sulfate reduction and anaerobic methane oxidation in Black Sea sediments. Deep-Sea Res. Part I-Oceanogr. Res. Pap. 48, 2097–2120. 10.1016/S0967-0637(01)00007-3

[B29] Kaden H. Peeters F. Lorke A. Kipfer R. Tomonaga Y. Karabiyikoglu M. (2010). Impact of lake level change on deep-water renewal and oxic conditions in deep saline Lake Van, Turkey. Water Resour. Res. 46:W11508 10.1029/2009WR008555

[B30] Kadioglu M. Sen Z. Batur E. (1997). The greatest soda-water lake in the world and how it is influenced by climatic change. Ann. Geophys. 15, 1489–1497. 10.1007/s00585-997-1489-9

[B31] Kallmeyer J. Ferdelman T. G. Weber A. Fossing H. Jørgensen B. B. (2004). A cold chromium distillation procedure for radiolabeled sulfide applied to sulfate reduction measurements. Limnol. Oceanogr. Methods 2, 171–180. 10.4319/lom.2004.2.171

[B32] Kallmeyer J. Grewe S. Glombitza C. Kitte J. A. (2015). Microbial abundance in lacustrine sediments: a case study from Lake Van, Turkey. Int. J. Earth Sci. 104, 1667–1677. 10.1007/s00531-015-1219-6

[B33] Kallmeyer J. Pockalny R. Adhikari R. R. Smith D. C. D'Hondt S. (2012). Global distribution of microbial abundance and biomass in subseafloor sediment. Proc. Natl. Acad. Sci. U.S.A. 109, 16213–16216. 10.1073/pnas.1203849109 22927371 PMC3479597

[B34] Kallmeyer J. Smith D. C. Spivack A. J. D'Hondt S. (2008). New cell extraction procedure applied to deep subsurface sediments. Limnol. Oceanogr. Methods 6, 236–245. 10.4319/lom.2008.6.236

[B35] Kempe S. Kazmierczak J. Landmann G. Konuk T. Reimer A. Lipp A. (1991). Largest known microbialites discovered in Lake Van, Turkey. Nature 349, 605–608. 10.1038/349605a0

[B36] Laanbroek H. J. Veldkamp H. (1982). Microbial interactions in sediment communities. Philos. Trans. R. Soc. Lond. B. Biol. Sci. 297, 533–550. 10.1098/rstb.1982.0059 6125961

[B37] Lin L.-H. Slater G. F. Sherwood Lollar B. Lacrampe-Couloume G. Onstott T. C. (2005). The yield and isotopic composition of radiolytic H_2_, a potential energy source for the deep subsurface biosphere. Geochim. Cosmochim. Acta 69, 893–903. 10.1016/j.gca.2004.07.032

[B38] Lipp J. S. Morono Y. Inagaki F. Hinrichs K.-U. (2008). Significant contribution of Archaea to extant biomass in marine subsurface sediments. Nature 454, 991–994. 10.1038/nature07174 18641632

[B39] Litt T. Anselmetti F. S. Baumgarten H. Beer J. Cagatay N. Cukur D. (2012). 500,000 Years of environmental history in eastern Anatolia: the PALEOVAN drilling project. Sci. Drill. 14, 18–29. 10.5194/sd-14-18-2012

[B40] Litt T. Krastel S. Sturm M. Kipfer R. Orcen S. Heumann G. (2009). ‘PALEOVAN’, International Continental Scientific Drilling Program (ICDP): site survey results and perspectives. Quat. Sci. Rev. 28, 1555–1567. 10.1016/j.quascirev.2009.03.002

[B41] Lloyd K. G. May M. K. Kevorkian R. T. Steen A. D. (2013). Meta-analysis of quantification methods shows that archaea and bacteria have similar abundances in the subseafloor. Appl. Environ. Microbiol. 79, 7790–7799. 10.1128/AEM.02090-13 24096423 PMC3837824

[B42] Long H. Flemings P. Germaine J. Saffer D. Dugan B. (2008). Data report: consolidation characteristics of sediments from IODP Expedition 308, Ursa Basin, Gulf of Mexico, in Proceedings of the IODP, 308, eds Flemings P. B. Behrmann J. H. John C. M. the Expedition 308 Scientists (College Station, TX: Integrated Ocean Drilling Program Management International Inc.).

[B43] Lyon E. J. Shima S. Buurman G. Chowdhuri S. Batschauer A. Steinbach K.. (2004). UV−A/blue−light inactivation of the ‘metal−free’hydrogenase (Hmd) from methanogenic archaea. Eur. J. Biochem. 271, 195–204. 10.1046/j.1432-1033.2003.03920.x 14686932

[B44] Manheim F. T. (1966). A hydraulic squeezer for obtaining interstitial water from consolidated and unconsolidated sediments. USGS Ref. Pap. 550, 171–174.

[B45] Martens C. S. Berner R. A. (1974). Methane production in the interstitial waters of sulfate-depleted marine sediments. Science 185, 1167–1169. 10.1126/science.185.4157.1167 17835470

[B46] Megonigal J. P. Hines M. E. Visscher P. T. (2005). Anaerobic metabolism: linkages to trace gases and aerobic processes, in Treatise on Geochemistry, eds Holland H. D. Turekian K. K. (Oxford: Pergamon), 317–424.

[B47] Middelburg J. J. (1989). A simple rate model for organic-matter decomposition in marine-sediments. Geochim. Cosmochim. Acta 53, 1577–1581. 10.1016/0016-7037(89)90239-1

[B48] Mitterer R. M. Malone M. J. Goodfriend G. A. Swart P. K. Wortmann U. G. Logan G. A. (2001). Co−generation of hydrogen sulfide and methane in marine carbonate sediments. Geophys. Res. Lett. 28, 3931–3934. 10.1029/2001GL013320

[B49] Morono Y. Terada T. Masui N. Inagaki F. (2009). Discriminative detection and enumeration of microbial life in marine subsurface sediments. ISME J. 3, 503–511. 10.1038/ismej.2009.1 19212428

[B50] Nealson K. H. (2005). Hydrogen and energy flow as “sensed” by molecular genetics. Proc. Natl. Acad. Sci. U.S.A. 102, 3889–3890. 10.1073/pnas.0500211102 15753283 PMC554801

[B51] Nealson K. H. Inagaki F. Takai K. (2005). Hydrogen-driven subsurface lithoautotrophic microbial ecosystems (SLiMEs): do they exist and why should we care? Trends Microbiol. 13, 405–410. 10.1016/j.tim.2005.07.010 16054814

[B52] Nickel J. C. Di Primio R. Mangelsdorf K. Stoddart D. Kallmeyer J. (2012). Characterization of microbial activity in pockmark fields of the SW-Barents Sea. Mar. Geol. 332, 152–162. 10.1016/j.margeo.2012.02.002

[B53] Nunoura T. Soffientino B. Blazejak A. Kakuta J. Oida H. Schippers A.. (2009). Subseafloor microbial communities associated with rapid turbidite deposition in the Gulf of Mexico continental slope (IODP Expedition 308). FEMS Microbiol. Ecol. 69, 410–424. 10.1111/j.1574-6941.2009.00718.x 19583789

[B54] Odom J. M. Peck H. D. Jr. (1984). Hydrogenase, electron-transfer proteins, and energy coupling in the sulfate-reducing bacteria Desulfovibrio. Annu. Rev. Microbiol. 38, 551–592. 10.1146/annurev.mi.38.100184.003003 6093686

[B55] Peters J. W. Schut G. J. Boyd E. S. Mulder D. W. Shepard E. M. Broderick J. B. (2015). [FeFe]- and [NiFe]-hydrogenase diversity, mechanism, and maturation. Biochi. Biophys. Acta 1853, 1350–1369. 10.1016/j.bbamcr.2014.11.021 25461840

[B56] Reimer A. Landmann G. Kempe S. (2009). Lake Van, eastern anatolia, hydrochemistry and history. Aquat. Geochem. 15, 195–222. 10.1007/s10498-008-9049-9

[B57] Røy H. Kallmeyer J. Adhikari R. R. Pockalny R. Jørgensen B. B. D'Hondt S. (2012). Aerobic microbial respiration in 86-million-year-old deep-sea red clay. Science 336, 922–925. 10.1126/science.1219424 22605778

[B58] Sagemann J. Jørgensen B. B. Greeff O. (1998). Temperature dependence and rates of sulfate reduction in cold sediments of Svalbard, Arctic Ocean. Geomicrobiol. J. 15, 85–100. 10.1080/01490459809378067

[B59] Sauer P. Glombitza C. Kallmeyer J. (2012). A system for incubations at high gas partial pressure. Front. Microbiol. 3:25. 10.3389/fmicb.2012.00025 22347218 PMC3271276

[B60] Schink B. Lupton F. S. Zeikus J. G. (1983). Radioassay for hydrogenase activity in viable cells and documentation of aerobic hydrogen-consuming bacteria living in extreme environments. Appl. Environ. Microbiol. 45, 1491–1500. 16346288 10.1128/aem.45.5.1491-1500.1983 PMC242490

[B61] Schrum H. N. Murray R. W. Gribsholt B. (2012). Comparison of Rhizon sampling and whole round squeezing for marine sediment porewater. Sci. Drill. 13, 47–50. 10.5194/sd-13-47-2012

[B62] Seeberg−Elverfeldt J. Schlüter M. Feseker T. Kölling M. (2005). Rhizon sampling of porewaters near the sediment−water interface of aquatic systems. Limnol. Oceanogr. Methods 3, 361–371. 10.4319/lom.2005.3.361

[B63] Soffientino B. Spivack A. J. Smith D. C. D'Hondt S. (2009). Hydrogenase activity in deeply buried sediments of the Arctic and North Atlantic Oceans. Geomicrobiol. J. 26, 537–545. 10.1080/01490450903104232

[B64] Soffientino B. Spivack A. J. Smith D. C. Roggenstein E. B. D'Hondt S. (2006). A versatile and sensitive tritium-based radioassay for measuring hydrogenase activity in aquatic sediments. J. Microbiol. Methods 66, 136–146. 10.1016/j.mimet.2005.11.004 16356571

[B65] Solheim A. (1991). The depositional environment of surging sub-polar tidewater glaciers: a case study of the morphology, sedimentation and sediment properties in a surge affected marine basin outside Nordaustlandet, Northern Barents Sea, Vol. 197 Norsk Polarinstitutt. Skrifter.

[B66] Spear J. R. Walker J. J. McCollom T. M. Pace N. R. (2005). Hydrogen and bioenergetics in the Yellowstone geothermal ecosystem. Proc. Natl. Acad. Sci. U.S.A. 102, 2555–2560. 10.1073/pnas.0409574102 15671178 PMC548998

[B67] Stevens T. O. McKinley J. P. (1995). Lithoautotrophic microbial ecosystems in deep basalt aquifers. Science 270, 450–454. 10.1126/science.270.5235.450

[B68] Teske A. P. (2012). Tracking microbial habitats in subseafloor sediments. Proc. Natl. Acad. Sci. U.S.A. 109, 16756–16757. 10.1073/pnas.1215867109 23047701 PMC3479487

[B69] Thamdrup B. Fleischer S. (1998). Temperature dependence of oxygen respiration, nitrogen mineralization, adn nitrification in arctic sediments. Aquat. Microb. Ecol. 15, 191–199. 10.3354/ame015191

[B70] Treude T. Boetius A. Knittel K. Wallmann K. Jørgensen B. B. (2003). Anaerobic oxidation of methane above gas hydrates at Hydrate Ridge, NE Pacific Ocean. Mar. Ecol. Prog. Ser. 264, 1–14. 10.3354/meps264001

[B71] Treude T. Niggemann J. Kallmeyer J. Wintersteller P. Schubert C. J. Boetius A. (2005). Anaerobic oxidation of methane and sulfate reduction along the Chilean continental margin. Geochim. Cosmochim. Acta 69, 2767–2779. 10.1016/j.gca.2005.01.002

[B72] Vignais P. M. Billoud B. (2007). Occurrence, classification, and biological function of hydrogenases: an overview. Chem. Rev. 107, 4206–4272. 10.1021/cr050196r 17927159

[B73] Vuillemin A. Ariztegui D. Team P. S. (2013). Geomicrobiological investigations in subsaline maar lake sediments over the last 1500 years. Quat. Sci. Rev. 71, 119–130. 10.1016/j.quascirev.2012.04.011

[B74] Wang G. Z. Spivack A. J. Rutherford S. Manor U. D'Hondt S. (2008). Quantification of co-occurring reaction rates in deep subseafloor sediments. Geochim. Cosmochim. Acta 72, 3479–3488. 10.1016/j.gca.2008.04.024

[B75] Widdel F. Rabus R. (2001). Anaerobic biodegradation of saturated and aromatic hydrocarbons. Curr. Opin. Biotechnol. 12, 259–276. 10.1016/S0958-1669(00)00209-3 11404104

[B76] Xie S. Lipp J. S. Wegener G. Ferdelman T. G. Hinrichs K.-U. (2013). Turnover of microbial lipids in the deep biosphere and growth of benthic archaeal populations. Proc. Natl. Acad. Sci. U.S.A. 110, 6010–6014. 10.1073/pnas.1218569110 23530229 PMC3625344

[B77] Zeikus J. G. (1977). The biology of methanogenic bacteria. Bacteriol. Rev. 41, 514–541. 329834 10.1128/br.41.2.514-541.1977 PMC414011

[B78] Zhabina N. N. Volkov I. I. (1978). A method of determination of various sulfur compounds in sea sediments and rocks, in Environmental Biogeochemistry and Geomicrobiology, ed Krumbein W. E. (Ann Arbor, MI: Ann Arbor Science), 735–745.

